# Quantitative Multiplexed Rapid Immunoprecipitation and Mass Spectrometry of Endogenous Proteins (qPLEX‐RIME) in Cell Lines

**DOI:** 10.1002/cpz1.70494

**Published:** 2026-10-31

**Authors:** Soleilmane Omarjee, Evangelia K. Papachristou, Chandra Sekhar Reddy Chilamakuri, Hamish McMillan, Kamal Kishore, Joshua Kent, Valar Nila Roamio Franklin, Clive D'Santos, Jason S. Carroll

**Affiliations:** ^1^ Cancer Research UK and Cambridge Institute University of Cambridge, Robinson Way Cambridge UK

**Keywords:** chromatin proteomics, endogenous protein‐protein interactions, qPLEX‐RIME, tandem mass tag, TMT labeling, quantitative mass spectrometry

## Abstract

Quantitative multiPLEXed Rapid Immunoprecipitation and Mass spectrometry of Endogenous proteins (qPLEX‐RIME) is a method for the quantitative discovery of endogenous protein complexes, without the need for genetically encoded affinity tags. It is particularly well suited to chromatin‐associated proteins and transcription factors, permitting rapid, robust, and quantitative identification of the protein components of a specifically purified complex directly from the endogenous protein of interest. The method uses sequential double fixation with disuccinimidyl glutarate (DSG) and formaldehyde to stabilize protein complexes and preserve low‐abundance or transient interactions. Target protein complexes are captured using magnetic‐bead‐coupled antibodies raised against the endogenous protein, and the resulting immunoprecipitates are washed extensively before on‐bead tryptic digestion. Resulting peptides from each experimental condition are labeled with isobaric tandem mass tags (TMT or TMTpro), pooled, and analyzed together in a single LC‐MS/MS run, allowing relative quantification of factor‐associated proteins across multiple conditions and biological replicates within one experiment. We further describe a complete bioinformatic workflow, using the qPLEXanalyzer R package, for processing raw peptide intensities into a statistically supported list of differentially enriched interacting proteins. Although the method depends on access to high‐resolution Tribrid mass spectrometry instrumentation and a highly specific antibody against the protein of interest, it represents a comparatively rapid and reproducible approach for the discovery of protein‐protein interactions, applicable across cell line models as well as patient‐derived xenograft and primary tumor tissue. © 2026 The Author(s). *Current Protocols* published by Wiley Periodicals LLC.

**Basic Protocol 1**: Sample processing and immunoprecipitation

**Basic Protocol 2**: Proteomic processing

**Basic Protocol 3**: Bioinformatic analysis

## INTRODUCTION

Protein complex discovery has been a longstanding goal in molecular biology because the composition of a complex reveals not only a protein's immediate binding partners but also how those interactions rewire in pathological conditions such as cancer. Co‐immunoprecipitation (co‐IP) using antibodies against a native, untagged protein of interest is among the oldest methods for isolating such complexes, dating to early protein A‐based adsorption methods (Kessler, 1975) and used to identify, for example, the p53‐SV40 T antigen complex directly from native cell lysate (Lane & Crawford, 1979). The principal limitation of co‐IP has always been its dependence on an antibody of sufficient specificity and affinity for the endogenous target, as suitable antibodies have historically been difficult and slow to generate for a large fraction of the proteome. Affinity purification combined with mass spectrometry (AP‐MS) using genetically encoded epitope tags, such as the tandem affinity purification (TAP) tag (Rigaut et al., 1999), emerged specifically to sidestep this antibody bottleneck: a small number of well‐characterized generic tag antibodies could be reused across any protein of interest, at the cost of requiring the target to be expressed from an introduced vector. This is a trade‐off rather than a strict improvement because exogenous expression can alter bait stoichiometry relative to endogenous levels and the tag itself can perturb localization or complex formation; native‐antibody approaches have therefore continued to be used at scale alongside tagged AP‐MS, including in systematic surveys of endogenous nuclear complexes comprising thousands of individual native immunoprecipitations (Malovannaya et al., 2011). The two approaches address different practical constraints (antibody availability versus tag‐induced artefact) rather than representing sequential generations of the same method, and both remain in active use today, including within this manuscript.

A separate challenge has been achieving statistically confident quantitative comparisons between conditions once a complex is isolated. This was first addressed by whole‐proteome metabolic labeling, specifically ^15^N labeling of one condition relative to another (Oda et al., 1999), later formalized as the stable isotope labeling by the amino acids in cell culture (SILAC) technique, in which a heavy‐ or light‐isotope‐labeled amino acid, typically leucine or arginine, is incorporated into one of two compared cell populations during culture (Ong et al., 2002). The isotope‐coded affinity tag (ICAT) method involves a complementary chemical approach, using hydrogen or deuterium to tag cysteine residues directly (Himeda et al., 2004). Both methods, however, are constrained either to cultured cells capable of metabolic labeling or to peptides containing cysteine, limiting their applicability to primary or clinical material, and both compare only two conditions per experiment.

To map endogenous protein complexes specifically in a chromatin context, we developed rapid immunoprecipitation and mass spectrometry of endogenous proteins (RIME; Mohammed et al., 2016), which combines native‐antibody co‐IP with formaldehyde cross‐linking adapted from chromatin immunoprecipitation (ChIP) methodology, on‐bead tryptic digestion to minimize antibody‐derived background, and SILAC‐based quantification. RIME is best understood not as a new class of method but as a chromatin‐optimized implementation of native‐antibody co‐IP‐MS: its formaldehyde cross‐linking step stabilizes transient chromatin‐associated interactions that would otherwise be lost during the stringent washing needed to remove the very high background typical of nuclear lysates, and its on‐bead digestion avoids the antibody and elution‐buffer contamination that complicates direct co‐IP‐MS of low‐abundance nuclear targets. The term “rapid” reflects the overall turnaround from starting sample to a list of candidate interactors, typically 2‐3 days, relative to the time required for complex purification and validation pipelines built around exogenous tagging and clonal cell line generation, rather than the duration of the immunoprecipitation step itself, which, like most native‐antibody co‐IPs, is run for several hours to overnight to maximize recovery of the target and its associated complex. Formaldehyde cross‐links primary amines that lie within ∼2‐3 Å of one another (Sutherland et al., 2008); this proximity requirement reduces, but does not eliminate, nonspecific cross‐linking, as formaldehyde also reacts with and neutralizes surface charges on proteins, which can itself alter native binding affinities and promote spurious associations rather than only suppressing them. RIME was first used to characterize the interactome of the estrogen receptor (Mohammed et al., 2013) and later to describe nuclear receptor cross‐talk between the estrogen and progesterone receptors in breast cancer (Mohammed et al., 2015), contributing to a wider chromatin proteomics field with a primary literature that substantially predates either paper (e.g., Vermeulen et al., 2010) and which has more recently been reviewed in depth (Van Mierlo et al., 2021). Cross‐linking efficiency was subsequently improved by combining disuccinimidyl glutarate (DSG) with formaldehyde in a dual‐cross‐linking strategy adapted from earlier ChIP methodology (Dufour et al., 2022; Nowak et al., 2005): DSG cross‐links primary amines directly between proteins, independently of DNA, capturing protein‐protein contacts within complexes that formaldehyde's protein‐DNA cross‐linking chemistry alone can under‐represent, particularly for more peripheral or transiently associated complex members.

qPLEX‐RIME, the method detailed in this article, replaces RIME's original SILAC‐based quantification with isobaric tandem mass tag (TMT) labeling applied after lysis (Papachristou et al., 2018). This is the central practical difference between the two methods: because TMT labeling does not require metabolic incorporation during live‐cell culture, it can be applied to material that cannot be labeled by SILAC, including patient‐derived xenograft tumors and primary human tumor tissue, and, being an isobaric rather than isotopic strategy, it allows multiple conditions to be pooled, resolved only during tandem mass spectrometry (MS/MS) fragmentation, and quantified within a single liquid chromatography‐tandem mass spectrometry (LC‐MS/MS) run, in contrast to the two‐condition comparisons SILAC permits. With the latest generation of TMTpro reagents, it is now possible to compare up to 18 different conditions in a single MS run (Li et al., 2021), substantially increasing the statistical power and experimental scope achievable in a single experiment relative to the original RIME method.

In this article, we describe a complete, updated qPLEX‐RIME workflow spanning from wet‐bench sample preparation through to bioinformatic interpretation of the resulting data (Fig. 1). Basic Protocol 1 describes double cross‐linking, chromatin shearing, and antibody‐mediated immunoprecipitation of the endogenous target protein complex from cultured cells. Basic Protocol 2 describes on‐bead tryptic digestion, TMT/TMTpro isobaric labeling, and high‐pH fractionation of the resulting peptides in preparation for LC‐MS/MS analysis. Basic Protocol 3 describes the downstream bioinformatic workflow, using the qPLEXanalyzer R package, for quality control, normalization, and statistical identification of differentially enriched, interacting proteins from the acquired mass spectrometry data.

**Figure 1:**
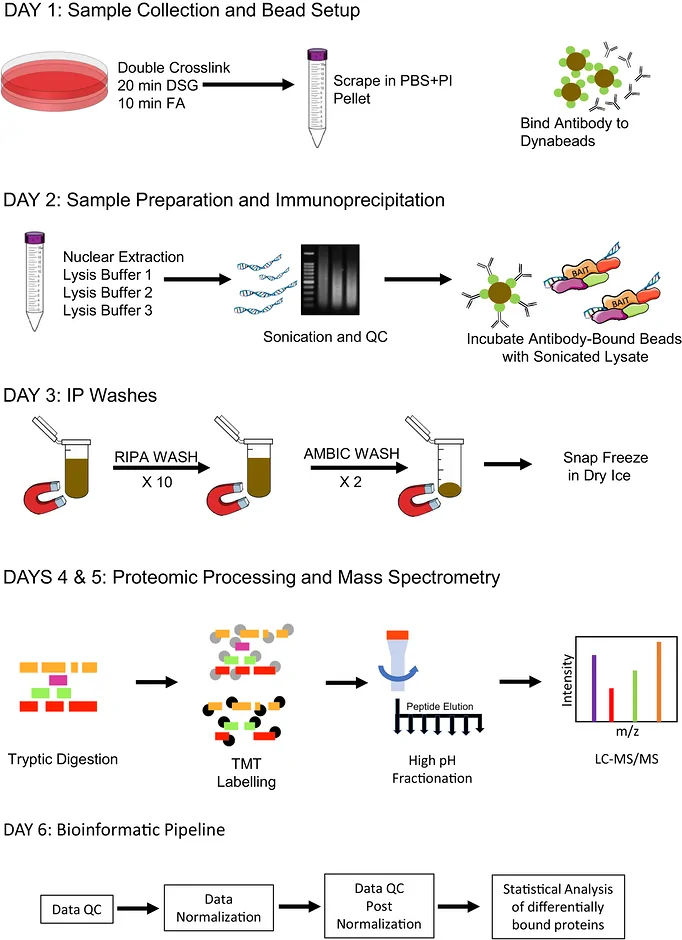
Schematic representation of the qPLEX‐RIME process.

## STRATEGIC PLANNING

### Biological Replicates

A minimum of four biological replicates per condition is recommended. This increases statistical power for the limma‐based differential analysis described in Basic Protocol 3 and reduces the risk that a single outlying replicate might produce an underpowered or misleading comparison.

### Bait Protein Abundance

Because qPLEX‐RIME includes a nuclear enrichment step, unequal starting levels of the bait protein between conditions can be mistaken for, or can mask, genuine differences in complex composition, and this is not something that data normalization can reliably correct for after the fact. We therefore strongly recommend verifying relative bait protein abundance across all conditions by western blot of the nuclear fraction before committing samples to a full pulldown.

### Antibody Validation and Negative Controls

The specificity of the antibody used against the endogenous target is the single largest determinant of data quality in this protocol, as RIME, unlike tagged AP‐MS approaches, has no generic affinity handle to fall back on. Antibody performance should be confirmed by western blotting, and ideally by a small‐scale pilot immunoprecipitation, before use in a full experiment. A matched negative control, typically a species‐ and isotype‐matched IgG pulldown processed in parallel, should be included for every condition to allow nonspecific background binders to be distinguished from genuine interactors during the statistical analysis in Basic Protocol 3.

### Instrument and Facility Scheduling

The complete qPLEX‐RIME workflow, from cell harvest to LC‐MS/MS acquisition, spans approximately one working week and depends on access to an Orbitrap Tribrid mass spectrometer for an extended, largely unattended overnight run (see Time Considerations). Because sample pools cannot be split once TMT labeled and combined, all conditions and replicates intended for a given experiment should be processed together, and mass spectrometry instrument time should be booked in advance of beginning the wet‐lab protocol.

### Safe Stopping Points

All safe stopping points noted within the protocol steps. Samples can be stored under the recommended conditions at each of these points, allowing the workflow to be split across multiple days without loss of sample integrity.

## Basic Protocol 1 SAMPLE PROCESSING AND IMMUNOPRECIPITATION

Basic Protocol 1 describes the wet‐bench workflow for capturing an endogenous protein complex from adherent cells in preparation for mass spectrometry analysis. Starting from cells at ∼80% confluency (15‐20 million cells from two 15‐cm dishes per pulldown), cells are sequentially cross‐linked with disuccinimidyl glutarate (DSG) and 1% formaldehyde to stabilize both direct and more peripheral protein‐protein interactions, quenched with glycine, and harvested in the presence of freshly prepared protease inhibitors (and phosphatase inhibitors, where phosphoprotein information is required). Cells are lysed stepwise and chromatin is fragmented by optimized sonication to a target size of 200‐300 bp, verified by gel electrophoresis (Fig. 2), and then antibody‐conjugated Dynabeads are used to immunoprecipitate the target protein complex overnight. Stringent bead washing removes nonspecifically bound material and prepares the sample for compatibility with downstream mass spectrometry. Throughout this protocol, particular attention should be paid to cross‐linking reagent freshness, sonication optimization, and antibody‐bead coupling (see Critical Parameters), as these determine both the specificity and reproducibility of the immunoprecipitated complex carried forward into Basic Protocol 2.

**Figure 2:**
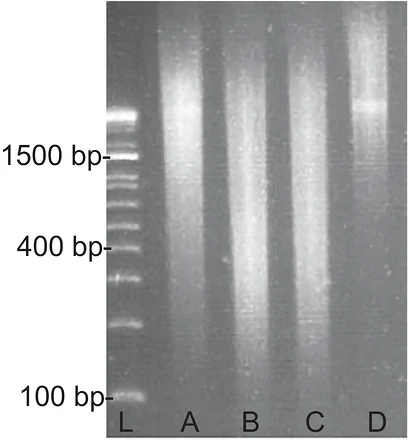
Sonication quality control. Four different MCF7 cell samples were sonicated 15 times using a 30 sec on/30 sec off pattern in a Bioruptor Plus Sonicator. The samples were then reverse cross‐linked at 95°C for 10 min and purified using a Qiagen PCR Purification column, and 20 µl of the eluate was run on a 2% E‐Gel. Lane L: 1 kb molecular weight ladder. Lanes B and C show sufficient sonication to proceed with the qPLEX‐RIME protocol. The sample in lane A would require 2‐3 further cycles of sonication, whereas lane D shows almost no sonication, so that this sample would need to be sonicated for 8‐10 extra cycles.

### Materials

Cells of interest growing in adherent culture in 15‐cm dishes
Phosphate‐buffered saline (PBS), room temperature and ice cold
Disuccinimidyl glutarate (DSG; Santa Cruz cat. no. sc‐285455A)
Dimethyl sulfoxide (DMSO; Sigma cat. no. 276855)
16% formaldehyde, methanol free (Thermo Scientific cat. no. 28908)
2.5 M glycine, pH 7.5 (Sigma cat. no. 5004)
cOmplete EDTA‐free Protease Inhibitors (PI; Sigma cat. no. 11873580001)
Phosphatase inhibitors (Thermo Scientific, cat. no. 78427)
Magnetic beads: Dynabeads Protein A/G (Thermo Fisher cat. no. 10001 or 10003)
5 mg/ml BSA in PBS (Sigma cat. no. A9418)
Lysis buffers 1, 2, and 3 (LB1, LB2, and LB3; see Table 1)
PCR purification kit (Qiagen cat. no. 28106)
Triton X‐100 (Sigma cat. no. 11332481001)
RIPA buffer (see Table 2)
Ammonium hydrogen carbonate (Sigma cat. no. 09830)
Agarose and gel casting equipment or E‐Gel (Thermo Scientific cat. no. G501802 or Invitrogen cat. no. G6465EU)

Tissue culture scrapers (Fisher Scientific cat no. 08‐100‐240)
Diagenode Bioruptor Plus Sonicator (Diagenode cat no. B01020014)
Refrigerated centrifuge capable of accommodating 1.5‐ml microcentrifuge (Eppendorf) tubes and 15‐ml Corning tubes
Magnetic stand (Invitrogen Dynamag 2 cat. no. 12321D)
Orbital shaker capable of accommodating 1‐liter conical flasks
Cold room, 4°C
Tube rotator (Stuart cat. no. UY‐07650‐11)
2‐ml Protein LoBind tubes (Eppendorf cat. no. 022431102)

**Table 1:** Preparation of Lysis Buffers 1, 2, and 3

**Component**	**Stock concentration**	**Final concentration**	**Vol. stock needed (for 100 ml)**	**Supplier and cat no**.
**Lysis Buffer 1 (LB1)** ***^a^***				
HEPES, pH 7.5	1 M	50 mM	5 ml	Fischer BP310‐1
NaCl	5 M	140 mM	2.8 ml	Fischer S/3160/65
EDTA	0.5 M	1 mM	0.2 ml	Acros 147850050
Glycerol	50% in ddH_2_O*^b^*	10%	20 ml	Fischer G/0650/17
NP‐40/Igepal	10%	0.5%	5 ml	Sigma 74385
Triton X‐100	10%	0.25%	2.5 ml	Fischer BP151/500
ddH_2_O			64.5 ml	
**Lysis Buffer 2 (LB2)** ***^a^***				
Tris•Cl, pH 8	1 M	10 mM	1 ml	Fischer BP152‐5
NaCl	5 M	200 mM	4 ml	Fischer S/3160/65
EDTA	0.5 M	1 mM	0.2 ml	Acros 147850050
EGTA	0.5 M	0.5 mM	0.1 ml	Sigma E3889
ddH_2_O			94.7 ml	
**Lysis Buffer 3 (LB3)** ***^a^***				
Tris•Cl, pH 8	1 M	10 mM	1 ml	Fischer BP152‐5
NaCl	5 M	100 mM	2 ml	Fischer S/3160/65
EDTA	0.5 M	1 mM	0.2 ml	Acros 147850050
EGTA	0.5 M	0.5 mM	0.1 ml	Sigma E3889
Sodium deoxycholate	10%	0.1%	1 ml	Sigma D6750
*N*‐Laurylsarcosine	20%	0.5%	2.5 ml	Sigma L9150
ddH_2_O			93.2 ml	

All three buffers may be stored up to 1 year at 4°C.

ddH_2_O, double‐distilled water.

**Table 2:** Preparation of RIPA Buffer

**Component**	**Final concentration**	**Supplier and cat. no**.
HEPES, pH 7.6	50 mM	Fischer BP310‐1
EDTA	1 mM	Acros 147850050
Sodium deoxycholate	0.7%	Sigma D6750
NP‐40	1%	Sigma 74385
LiCl	0.5 M	Acros 19988530
Double‐distilled water (ddH_2_O)		

RIPA buffer may be stored up to 1 year at 4°C.

#### Sample processing

1 Culture cells of interest to 80% confluency in 15‐cm dishes, such that two dishes will provide the ∼15‐20 million cells needed per pulldown.
  Formaldehyde and disuccinimidyl glutarate (DSG) solutions for cross‐linking should be made fresh at this point. Formaldehyde should be diluted in PBS to 1% final.
  DSG is very insoluble in PBS and should first be dissolved to 0.5 M in DMSO. This DMSO/DSG solution should then be added dropwise to PBS using a 200‐µl pipet under constant agitation with an orbital shaker to achieve a final concentration of 2 mM (allow for 20 ml per dish). Inspect the DSG solution to ensure that it is free from any precipitates. In the event of precipitate formation, start over again with a fresh stock of DSG/DMSO.
2 Discard culture medium from tissue culture dishes, add 20 ml PBS/2 mM DSG (room temperature) to the cells on the dish, and incubate at room temperature for 20 min.
3 Discard PBS/2 mM DSG solution and add 20 ml PBS/1% formaldehyde. Incubate at room temperature for 10 min.
4 Quench by adding a 1:10 volume of 2.5 M glycine, pH 7.5, and incubate at room temperature for at least 3 min.
  A separate PBS wash between removing culture medium and adding the DSG/formaldehyde cross‐linking solutions is not included, as residual medium retained on the cell monolayer after aspiration is negligible in volume relative to the cross‐linker solutions added (20 ml PBS/2 mM DSG, then 20 ml PBS/1% formaldehyde, per dish) and is not sufficient to meaningfully quench the cross‐linker or introduce medium‐derived contaminants into the downstream mass spectrometry data. Users working with unusually protein‐rich or serum‐heavy culture medium, or scaling the protocol down substantially, may wish to include a brief PBS rinse at this point as a precaution.
5 Pour the quenched formaldehyde solution and wash the cells twice with ice‐cold PBS. If processing several plates at the same time, leave the cells in the second PBS wash and store them at 4°C.
6 Prepare a fresh solution of protease inhibitors by dissolving two tablets of cOmplete EDTA‐free protease inhibitors in 1 ml PBS (100× stock solution). Keep this solution on ice and add to buffers as required.
  Phosphatase inhibitors should be added to the pulldown buffers if phosphoprotein information is required.
7 Prepare 1 ml of PBS containing protease inhibitors per dish to be harvested by diluting the 100× stock from step 6 into the appropriate volume of PBS. Pour off and aspirate excess PBS from the cells, add 1 ml PBS+protease inhibitors, and scrape cells using tissue culture scrapers.
8 Transfer all the cells to a 1.5‐ or 2‐ml microcentrifuge tube or a 15‐ml Falcon tube, depending on the number of dishes processed.
9 Centrifuge 5 min at 500 × *g*, 4°C, and remove all supernatant.
  Safe stopping point: Samples can be snap frozen and stored at –80°C at this point.
  Antibody needs to be bound to beads either at 4°C for 24 hr before the IP procedure or on the same day at room temperature for 3 hr.

#### Immunoprecipitation

10 Choose appropriate Dynabeads coupled with either protein A or G, depending on antibody isotype (https://uk.neb.com/tools‐and‐resources/selection‐charts/affinity‐of‐protein‐ag‐for‐igg‐types‐from‐different‐species)
11 Vortex beads and add 50 µl of Dynabeads (for sample material derived from two 15‐cm dishes of starting cells) to a round‐bottom 2‐ml microcentrifuge tube. Place tube on a magnetic stand to collect the magnetic beads.
12 Remove supernatant and wash beads three times with cold PBS+5 mg/ml BSA, using the magnet between steps to collect the magnetic beads.
13 Resuspend beads in 500 µl PBS+5 mg/ml BSA, add 5 µg antibody (for two 15‐cm dishes), and rotate overnight in the cold room at ∼12 rpm.
  Using 5 µg of antibody per IP (two 15‐cm dishes) is a validated starting point for standard IgG‐format antibodies, but is not a fixed requirement and should be titrated for each new antibody because the optimal amount varies with antibody affinity, format, and host species. A simple titration (e.g., 2, 5, and 10 µg) evaluated by western blot of the bead eluate against input is recommended when using a new antibody for the first time.
14 If cells were frozen after step 9, thaw cell pellets on ice.
15 Add 2 ml cold LB1 supplemented with 1× protease inhibitors (for two dishes) and resuspend. Rotate for 10 min at ∼15 rpm in the 4°C cold room and then centrifuge for 5 min at 2000 × *g*, 4°C.
16 Discard all supernatant and resuspend the pellet in 2 ml cold LB2 supplemented with 1× protease inhibitors (for two dishes). Rotate for 5 min at ∼15 rpm in the 4°C cold room and then centrifuge for 5 min at 2000 × *g*, 4°C.
17 Discard all supernatant, resuspend the pellet in 600 µl cold LB3 supplemented with 1× protease inhibitors (for two dishes), and transfer the solution to an adequate polystyrene sonication tube
18 Sonicate the samples at high intensity, 30 sec on, 30 sec off, for 15 cycles using a Bioruptor Plus water‐cooled sonicator.
  Sonication is a crucial step in the RIME process. Under‐sonication will lead to nonspecific pulldown, whereas over‐sonication can overheat the sample and subsequently affect pulldown efficiency. The above sonication procedure is given as an example only and should be optimized for every cell line by running a DNA gel to verify efficient sonication as described in step 19.
19 Take a 10‐µl aliquot of sonicated chromatin and resuspend it in 90 µl nuclease‐free water. Reverse cross‐link for 10 min at 95°C in a heat block. Use a Qiagen PCR Purification Kit to extract the sonicated chromatin, elute in 30 µl nuclease‐free water, and run 20 µl of the eluate on a 2% E‐Gel or 2% agarose gel (Fig. 2).
  This step is used to confirm sufficient sonication; fragment sizes should ideally be between 200 and 300 bp (Fig. 2).
20 After confirming that the sonication was sufficient, add 66 µl of 10% Triton X‐100 dissolved in LB3 to the sonicated lysate from step 18 (to a final concentration of 1% Triton X‐100). Mix by pipetting and centrifuge for at least 10 min at 20,000 × *g*, 4°C.
21 While the lysate is being centrifuged, wash the beads from step 13 three times with 1 ml PBS+5 mg/ml BSA each time, using the magnet to collect the beads between washes, and then resuspend beads in 200 µl LB3/PI/1% Triton X‐100.
22 Collect the supernatant from step 20 in a fresh 2‐ml Protein LoBind tube and top up the volume to 1.5 ml with LB3/PI/1% Triton X‐100.
23 Combine the beads and the chromatin and rotate overnight in the cold room at ∼12 rpm.
24 Wash the beads with 1 ml ice‐cold RIPA buffer 10 times, using the magnet to collect the beads between washes. It is important not to let the beads dry out at this stage.
  Do not supplement the RIPA buffer with protease inhibitors at this point as that can interfere with subsequent tryptic digestion.
25 Wash the beads twice with 1 ml freshly prepared 100 mM ammonium bicarbonate (AMBIC). During the first wash, transfer the beads to a fresh Protein LoBind tube for the second AMBIC wash.
26 Remove AMBIC and snap freeze the beads in dry ice.
  The washing steps need to be performed rapidly. To ensure removal of all AMBIC solution, proceed with a brief spin‐down after aspiration of the second AMBIC wash, place the tubes back on the magnet, and remove all residual AMBIC solution using a 20‐µl pipet.
  This is a safe stopping point. Samples can be stored long term at –80°C at this point.

## Basic Protocol 2 PROTEOMICS PROCESSING

Basic Protocol 2 converts the bead‐bound protein complexes recovered in Basic Protocol 1 into isobarically labeled peptides ready for mass spectrometry acquisition. All steps are performed in Protein LoBind tubes to minimize peptide loss at these low input amounts. Immunoprecipitated proteins undergo sequential on‐bead trypsin digestion in ammonium bicarbonate, after which peptides are acidified, desalted on C18 spin columns, and dried. Peptides from each condition are then labeled with TMT‐10plex (or TMTpro, if greater multiplexing is required), pooled into a single sample, and fractionated by high‐pH reverse‐phase chromatography to reduce complexity ahead of LC‐MS/MS. Fractions are reconstituted and analyzed by nanoLC‐MS/MS on an Orbitrap‐based Tribrid mass spectrometer operated in data‐dependent acquisition mode with synchronous precursor selection‐triple stage MS (SPS‐MS3) for accurate reporter‐ion quantification, and the resulting spectra are processed in Proteome Discoverer software using the SequestHT search engine under a 1% peptide‐level false discovery rate. Only high‐confidence, unique peptide abundances are exported from this protocol, forming the input data for the bioinformatic analysis described in Basic Protocol 3.

Notably, cross‐links are not reversed before trypsin digestion, and no reduction or alkylation step is included, consistent with standard practice for on‐bead digestion of formaldehyde/DSG‐cross‐linked immunoprecipitates. Trypsin retains access to cleavage sites on the non‐cross‐linked majority of the protein backbone, as cross‐linking is sub‐stoichiometric and localized to a minority of surface‐exposed lysine residues; protein identification, which relies on multiple peptides per protein, is therefore not substantially impaired, though peptides spanning a cross‐linked residue itself may be under‐represented. Reduction and alkylation, routinely used to resolve disulfide bonds in secreted or membrane proteins, is omitted because nuclear and chromatin‐associated proteins, the principal targets of this protocol, are predominantly found in the disulfide‐poor reducing environment of the nucleoplasm; users applying this protocol to targets with known disulfide‐bonded domains should consider adding a standard DTT/iodoacetamide step before digestion. Of important note, we found that reduction and alkylation of RIME samples causes a significant rise in immunoglobulin PSM, cutting the number of specific and nonspecific proteins identified by >50%.

### Materials

Protein complex of interest, bound to magnetic beads (Basic Protocol 1)
Ammonium hydrogen carbonate (Sigma cat. no. 09830)
Trypsin (Pierce, cat. no. 90058)
Formic acid (Fischer A117‐50)
Ultra‐Micro C18 SpinColumns (Harvard Apparatus cat. no. 74‐7206)
Triethylammonium bicarbonate (TEAB; Sigma cat. no. T4708)
TMT 10‐Plex or TMTpro 16‐plex reagent (Thermo Scientific cat. nos. 90406 and A44520)
Hydroxylamine (Thermo Scientific, cat. no. 90115)
Reverse‐phase cartridges (Pierce, cat. no. 84868)

1.5‐ml Protein LoBind tubes (Eppendorf cat. no. 022431081)
Vacuum concentrator centrifuge (Eppendorf E5305000568)
Equipment for mass spectrometry: for example,
  Dionex Ultimate 3000 UHPLC (Thermo Scientific)
  Orbitrap‐based Tribrid mass spectrometer, such as the Fusion Lumos (Thermo Scientific)
  EASY‐Spray analytical column, 75 µm × 25 cm, C18, 2 µm, 100 Å (Thermo Scientific cat. no. ES902)
  Acclaim PepMap 100 trapping column, 100 µm × 2 cm C18, 5 µm, 100 Å (Thermo Scientific cat. no. 164946)
Proteome Discoverer 2.4 software

*NOTE*: Protein LoBind tubes must be used for all steps below.

#### Sample preparation and liquid chromatography‐tandem mass spectrometry (LC‐MS/MS)

1 Thaw the stored beads at room temperature.
2 Prepare a solution of 15 ng/µl trypsin in 100 mM AMBIC. Add 10 µl of the solution to the beads and incubate overnight at 37°C to allow on‐bead digestion of proteins.
3 The next day, repeat the digestion step by freshly preparing and adding another 10 µl of trypsin solution and incubating the beads for 4 hr at 37°C.
4 Place the samples in a magnetic rack and collect supernatant in clean tubes. Acidify the supernatant by adding formic acid to 0.5% final.
5 Load the acidified peptide samples into Ultra‐Micro C18 SpinColumns and perform purification following the manufacturer's recommendations.
6 After elution, dry the peptide samples using a centrifugal concentrator.
  Safe stopping point: Samples can be stored at –20°C at this point.
7 Reconstitute the dried samples in 100 µl of 0.1 M TEAB.
8 Label each sample by adding 0.4 mg of TMT‐10plex reagent to each and incubating for 1 hr at room temperature.
  The multiplexity can be increased through the use of the TMTpro reagents. For TMTpro labeling, samples are reconstituted in 25 µl of 0.1 M TEAB, and 0.25 mg TMTpro is used to label each sample.
9 Pool all the labeled samples and dry the TMT mix using a centrifugal concentrator at 60°C.
  Safe stopping point: The TMT mix can be stored long term at –20°C or –80°C at this point before LC‐MS/MS analysis is performed.
10 Fractionate the TMT mix using reverse‐phase cartridges at high pH following the manufacturer's recommendations. Collect nine fractions using a range of 5%, 10%, 12.5%, 15%, 17.5%, 20%, 22.5%, 25%, and 50% acetonitrile in 0.1% triethylamine.
11 Reconstitute each fraction in 25 µl of 0.1% formic acid for liquid chromatography‐tandem mass spectrometry (LC‐MS/MS).
12 Analyze the peptide fractions using a process appropriate for your LC‐MS/MS apparatus. For example, with a Dionex Ultimate 3000 UHPLC System Coupled with the Nanos ESI Fusion Lumos (Thermo Scientific), use the following procedure:
  a. Load 5 µl of each fraction on the Acclaim PepMap 100 (100 µm × 2 cm C18, 5 µm, 100 Å) trapping column with the µlPickUp injection method using the loading pump at a 5 µl/min flow rate for 10 min.
  b. For peptide separation, use an EASY‐Spray analytical column (75 µm × 25 cm, C18, 2 µm, 100 Å) for multi‐step gradient elution.
  c. Use 2% acetonitrile/0.1% formic acid as mobile phase A and 80% acetonitrile/0.1% formic acid as mobile phase B.
  d. Elute at flow rate 300 nl/min, with the column maintained at 40°C throughout, as follows: 95 min, gradient up to 45% B; 5 min, gradient up to 95% B; 8 min, isocratic 95% B; 2 min, gradient down to 5% B; 10 min, isocratic equilibration in 5% B.
  e. Operate the Lumos mass spectrometer in positive‐ion mode with data‐dependent acquisition for both MS2 and SPS‐MS3 methods.
  f. Acquire full scans in the Orbitrap Tribrid with an *m*/*z* range of 380‐1500 at 120K resolution.
  g. Set an automatic gain control (AGC) target of 3.0e5 with a maximum injection time of 100 ms. Acquire MS2 scans in the ion trap with collision energy 35% and an isolation window 0.7 Th.
  h. Select the 10 most intense fragments for Synchronous Precursor Selection (SPS) HCD‐MS3 analysis with MS2 isolation window 2.0 Th. MS3 data are acquired in the Orbitrap at a resolution of 50K with a *m*/*z* scan range of 110–400. The higher‐energy collisional dissociation (HCD) collision energy is set at 55%.
    These details are applicable only when using the specific LC‐MS/MS equipment described in the hardware section and will need to be optimized for different equipment.

#### Data processing

13 Process the collected collision‐induced dissociation (CID) tandem mass spectra with the SequestHT search engine using Proteome Discoverer 2.4 software for peptide and protein identifications. For example:
  a. In our work, the processing workflow uses the Sequest HT node with a precursor mass tolerance of 20 ppm and a fragment mass tolerance of 0.5 Da, including dynamic modifications of oxidation of methionine (M, +15.995 Da) and deamidation of asparagine and glutamine (N, Q, +0.984 Da), and static modifications of TMT6plex at any N‐terminus or lysine residues (K, +229.163 Da).
  b. The Reporter Ion Quantifier node in the processing workflow was configured with activation type HCD, an integration tolerance of 20 ppm, integration method set to Most Confident Centroid, and MS order set to MS3.
  c. In the consensus workflow, the Reporter Ions Quantifier node used signal‐to‐noise (S/N) reporter quantification with Quan Value Corrections applied (True), a co‐isolation threshold of 100, an average reporter S/N threshold of 3, and an SPS mass matches threshold of 50%, followed by a Peptide Validator node with a strict target peptide FDR of 0.01.
    At the end of data processing, non‐normalized intensities (abundances) only for unique peptides identified with high confidence (FDR<1%) are exported for downstream analysis.

## Basic Protocol 3 BIOINFORMATIC ANALYSIS

Basic Protocol 3 describes the computational workflow for converting the peptide‐level intensity data generated in Basic Protocol 2 into a statistically supported list of differentially enriched interacting proteins, outlined schematically in Figure 3. All analysis is performed in R and RStudio (Linux, macOS, or Windows) using the tidyverse, statmod, and qPLEXanalyzer packages. Protein sequence annotation is first derived from reviewed UniProt (Swiss‐Prot) entries to generate an organism‐specific protein‐to‐gene mapping table. Peptide intensities and sample metadata are then imported and converted into an MSnSet object, within which quality control, median‐based normalization (GroupScaling), and peptide‐to‐protein summarization are performed. Differential enrichment between experimental groups is assessed using limma‐based statistical modelling (see Statistical Analysis), with results visualized as MA and volcano plots and exported as a final differential enrichment table. Only high‐confidence, unique peptide‐derived protein intensities are retained throughout, ensuring that the biological interpretation described under Understanding Results is based on robust and reproducible quantitative data.

**Figure 3:**
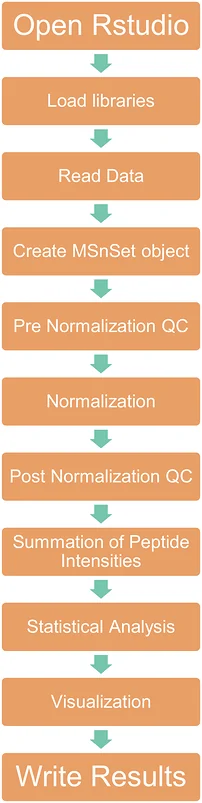
Schematic of the computational workflow used to convert peptide‐level intensity data into a statistically supported list of differentially enriched interacting proteins.

### Hardware

Computers running Linux, macOS, or Windows

#### Software

**Table:** 

R	https://www.r‐project.org/
RStudio	https://rstudio.com/
R tidyverse packages	https://www.tidyverse.org/; tidyverse can also be installed in R using the following command: install.packages("tidyverse")
Statmod	https://cran.r‐project.org/web/packages/statmod/index.html; Statmod can also be installed in R using the following command: install.packages("statmod")
qPLEXanalyzer	https://www.bioconductor.org/packages/release/bioc/html/qPLEXanalyzer.html; qPLEXanalyzer can also be installed in R using the following command: if (!requireNamespace("BiocManager", quietly = TRUE)){install.packages("BiocManager")} BiocManager::install("qPLEXanalyzer")

#### Input Files

**Table:** 

*Swiss_Prot_Homo_sapiens.fasta*	Obtained from https://www.uniprot.org/. This file contains Swiss‐Prot canonical protein sequences and is used for protein accession and gene symbol mapping; it will differ between organisms. The method for preparing this file is described in steps 2 and 3.
*human_protein_gene_mapping.txt*	This file is generated from the above fasta file using proteinID_and_gene_name_mapping.Rscript. It is a tab‐separated text file with three columns: Protein Accession, Gene Symbol, and Gene Description. This file can be reused for different analyses if the same organism is used (e.g., *Homo sapiens*).
*peptide_intensities.csv*	This file is typically derived from the Proteome Discoverer Software. It is a .csv file containing the peptide intensities for each sample. Each row is a peptide and each column a sample.
*sample_sheet.csv*	This .csv file contains all the metadata information and must contain at least five mandatory columns: SampleName: Column listing unique sample names SampleGroup: Column specifying the group the sample belongs to BioRep: Column containing the replicate number TechRep: Column containing information about whether the sample is a technical replicate Run: Column containing the mass spectrometer run number

#### Preparation of the protein data

Data from Proteome Discoverer typically have Protein IDs. For better functional interpretation in differential analysis, it is useful to map Protein IDs with gene names and any other relevant information, such as gene function. One way of achieving this is to download genome‐specific protein data from the UniProt database.

1 Go to https://www.uniprot.org/, and under UniProtKB, search for the genome of interest (e.g., human).
2 Open RStudio and load the required libraries as shown below:

library(tidyverse)
library(statmod)
library(qPLEXanalyzer)

3 Load peptide intensities, sample metadata, and annotation files:

sample_sheet <- read_csv('sample_sheet.csv')
intensities <- read_csv('peptide_intensities.csv')
annotation <- read_delim('human_protein_gene_mapping.txt', delim ='\t', col_names = T)

4 The qPLEXanalyzer software only works with MSnSet data structure which holds MS proteomics intensities data and the experimental meta‐data. Therefore, convert the data structure to MSnSet using the following command:


MSnset_int <- convertToMSnset(ExpObj = intensities, metadata = sample_sheet, Accessions =1, Sequences =2, indExpData = c(4:ncol(intensities)), rmMissing = TRUE)

5 At this step, it is essential to control the data quality. Various functions from qPLEXanalyzer described in Table 3 (some also illustrated in Figs. 4 and 5 *)* can help with quality analysis.

**Table 3:** Quality Control Assessment Measures

Intensity distribution plot (Supporting Information Fig. 1)	Within a biological group, intensities should be similar. An intensity plot can be generated using the command: intensityPlot(MSnset_int)
Intensity boxplot (Fig. 4)	These intensities can also be viewed in the form of boxplots: intensityBoxplot(MSnset_int)
Correlation plot (Supporting Information Fig. 2)	A correlation plot can be generated to visualize the level of association between samples and between groups using the command: corrPlot(MSnset_int, addValues = FALSE)
Hierarchical clustering (Supporting Information Fig. 3)	A dendrogram displaying hierarchical clustering can be produced using the command: hierarchicalPlot(MSnset_int)
PCA plot (Fig. 5)	A PCA plot displays the samples in a 2D plane based on their first two principal components. This is a good way of visualizing how replicates and groups differ from each other. pcaPlot(MSnset_int)
Coverage plot (Supporting Information Fig. 4)	The coverage plot displays the location of peptides identified across a protein sequence—in this example, the estrogen receptor (ER). The canonical fasta file (*P03372.fasta*) for the bait protein needs to be downloaded from the UniProt database (Supporting Information Fig. 11). This plot also displays the number of peptides identified and the percentage coverage, thereby providing a way to assess the efficiency of the immunoprecipitation. coveragePlot(MSnset_int, ProteinID = "P03372", ProteinName = "ESR1", fastaFile = 'P03372.fasta' )

**Figure 4:**
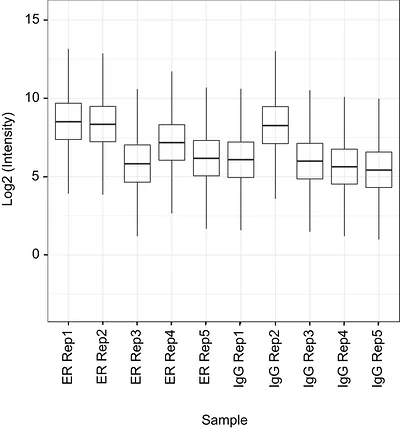
Example of an intensity boxplot generated during quality control of data from Basic Protocol 3.

**Figure 5:**
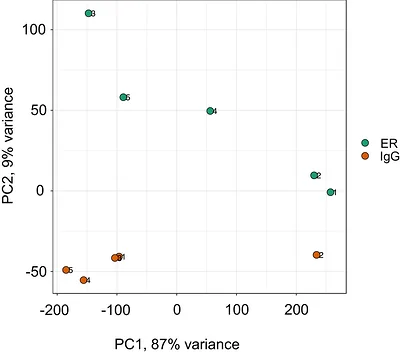
Example of a principal component analysis (PCA) plot generated during quality control of data from Basic Protocol 3.

#### Data normalization

Normalization is used to remove any technical artefacts accumulated during the experimental process which improves downstream analysis. qPLEXanalyzer provides three possible normalization methods—quantile scaling, median scaling, and row scaling—but median scaling is most commonly used. In qPLEXanalyzer, the GroupScaling function is used for the above median scaling method within a group.

6 Run the GroupScaling function using the following command:


MSnset_norm <- groupScaling(MSnset_int, scalingFunction = median, groupingColumn = "SampleGroup")

  Normalization will remove technical artifacts from the data, and it is always useful to verify the effectiveness of this normalization by visualizing the data using commands similar to those listed in step 5.
7 Visualize the normalized data using the commands below:
  a. Intensity distribution plot after normalization (Supporting Information Fig. 5):


intensityPlot(MSnset_norm)

  b. Intensity boxplot after normalization (Fig. 6):


intensityBoxplot(MSnset_norm)

  c. Correlation plot after normalization (Supporting Information Fig. 6):


corrPlot(MSnset_norm, addValues = FALSE)

  d. Hierarchical clustering after normalization (Supporting Information Fig. 7):


hierarchicalPlot(MSnset_norm)

  e. Principal component analysis (PCA) plot after normalization (Supporting Information Fig. 8):


pcaPlot(MSnset_norm)

**Figure 6:**
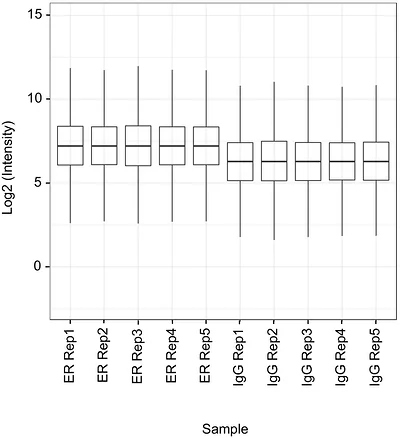
Example of a normalized intensity boxplot generated during quality control of data from Basic Protocol 3.

#### Summation of peptide intensities

Up until this point, the intensities worked with are peptide intensities, and each protein is represented by one or more peptide intensities. Ultimately, biological inferences will be made at the protein level, therefore these peptide intensities will have to be aggregated into protein intensities. For this aggregation, one can use either Sum, Mean, or Median, but Sum is the recommended method.

8 Aggregate the peptide intensities into protein intensities by using the SummarizeIntensities command below:


MSnset_protein <- summarizeIntensities(MSnset_norm, sum, annotation)

#### Statistical analysis for differentially bound proteins

qPLEXanalyzer uses a limma‐based analysis that has been developed and is used extensively for microarray data analysis. A linear model is fitted for each protein and then log_2_(fold change) values between comparisons are estimated.

9 Create the contrast between groups of interest using the command below, adjusted to represent the biological groups of interest (in this example, the two biological groups compared are an estrogen receptor (ER) pulldown and an IgG pulldown):


contrast <- c(ER_vs_IgG = "ER - IgG")

10 Compute the differential statistics between the two groups using the command:


diffstats <- computeDiffStats(MSnset_protein, contrasts = contrast)

11 Generate the differential expression table using:


de_table <- getContrastResults(diffstats = diffstats, contrast = contrast)

12 Generate a ratio intensity MA plot (Supporting Information Fig. 9) using:


maVolPlot(diffstats, contrast = contrast, plotType = "MA")

13 Generate a volcano plot (Fig. 7) using:


maVolPlot(diffstats, contrast = contrast, plotType = "Volcano")

14 Finally, write the results to a .csv file using:


write_csv(x=de_table, file = 'de_results.csv')

**Figure 7:**
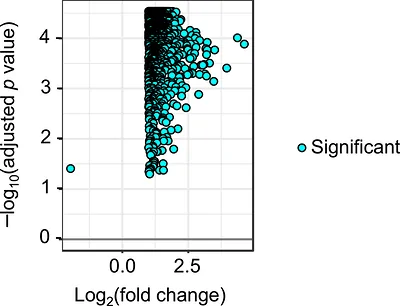
Example of a volcano plot generated for the statistical analysis of differentially bound proteins from Basic Protocol 3.

## COMMENTARY

### Background Information

This section focuses on the practical advantages and limitations of qPLEX‐RIME as implemented at the bench, its documented applications, and the chemistry underlying its key labeling and digestion steps.

The principal practical advantage of qPLEX‐RIME over tagged AP‐MS approaches is that it requires no genetic manipulation of the cell line or tissue under study, making it directly applicable to primary tumor material and patient‐derived xenografts, for which introducing an exogenous construct is either impractical or would itself confound the biology under investigation. Relative to the original SILAC‐based RIME method, qPLEX‐RIME's use of post‐lysis isobaric labeling removes the requirement for metabolic labeling during live‐cell culture, extending the method to any sample type, and its multiplexing capacity (up to 18 channels with TMTpro) allows considerably more conditions and replicates to be compared within a single, directly comparable mass spectrometry run. The principal limitation remains the method's dependence on the availability of a single, highly specific antibody against the endogenous target: unlike tagged AP‐MS, there is no generic affinity handle to fall back on if a suitable antibody does not exist, and antibody performance must be validated empirically for each new target (see Critical Parameters). A further limitation, shared with all cross‐linking‐based approaches, is that formaldehyde and DSG cross‐linking captures a snapshot of complex composition at a single, chemically defined moment and cannot resolve the dynamic assembly or disassembly kinetics of a complex over time.

qPLEX‐RIME's relationship to proximity‐labeling methods such as BioID and APEX is better understood as complementary rather than competing. Both BioID and APEX were originally developed as genetically encoded fusion proteins, but more recent “off‐the‐shelf” implementations deliver the labeling enzyme directly via a native antibody rather than a genetic fusion (Santos‐Barriopedro et al., 2021), making proximity labeling similarly applicable to unmodified, endogenous samples. The key distinction that remains is biochemical rather than genetic: proximity labeling biotinylates any protein within a several‐nanometer radius of the antibody‐enzyme conjugate over a defined labeling window, capturing a broader, more spatially defined neighborhood that can include indirect or transient neighbors, whereas qPLEX‐RIME's cross‐linking‐based capture favors proteins in direct or near‐direct contact with the bait, at the cost of being less able to resolve subcomplex spatial organization. The two approaches are increasingly used together to cross‐validate candidate interactors identified by either method alone.

qPLEX‐RIME has been used to track temporal changes in the estrogen receptor alpha (ERα) interactome following tamoxifen treatment in breast cancer cells, and to identify ERα‐associated proteins directly in patient‐derived xenograft tumors and primary human breast cancer tissue (Papachristou et al., 2018), demonstrating utility in monitoring both drug response and endogenous complex composition in clinical‐grade material. At the biochemical level, TMT and TMTpro reagents are amine‐reactive *N*‐hydroxysuccinimide (NHS) esters that react predominantly with peptide N‐termini and the ε‐amino group of lysine side chains; because trypsin cleaves C‐terminal to lysine and arginine residues, and TMT‐labeled lysines are refractory to efficient trypsin cleavage at that site, a modest increase in missed cleavages is an expected consequence of labeling rather than a digestion failure, and search parameters (Basic Protocol 2) account for this by setting TMT modification as a static modification during database searching.

### Critical Parameters

The success of qPLEX‐RIME depends on several factors that must be controlled at each stage of the workflow. In Basic Protocol 1, cross‐linking reagents must be prepared fresh immediately before use: formaldehyde degrades in solution over time, and DSG, being poorly soluble in aqueous buffer, must first be dissolved in DMSO and then added dropwise under constant agitation to avoid precipitation; any visible precipitate indicates that the DSG stock should be discarded and remade rather than used, as incomplete dissolution reduces the effective cross‐linker concentration and compromises complex stabilization. Bait protein abundance should also be verified by western blotting following nuclear fractionation before samples are committed to a full experiment because unequal starting levels of the protein of interest between conditions will confound downstream quantitative comparisons and cannot be corrected for through data normalization alone. Antibody amount and binding time are similarly critical: 5 µg of antibody per 2 × 15‐cm‐dish pulldown is a validated starting point for standard IgG‐format antibodies, but should be titrated for each new antibody (see annotation following Basic Protocol 1, step 13), as the optimal amount varies with antibody affinity, format, and host species. Sonication is one of the most sensitive steps in the protocol: under‐sonication leaves chromatin insufficiently fragmented and reduces immunoprecipitation efficiency, whereas over‐sonication can heat the sample and degrade complex integrity; because required sonication settings vary between cell lines and instruments, fragment size (200‐300 bp) must be verified by gel electrophoresis for every new cell line rather than assumed from prior experiments. Where phosphoprotein information is required, phosphatase inhibitors must be included in all buffers from the point of harvesting onward, as their omission cannot be corrected retrospectively.

In Basic Protocol 2, all sample handling should be performed in Protein LoBind tubes, as standard polypropylene tubes can result in substantial peptide loss at the low input amounts typical of endogenous pulldowns. Digestion completeness is dependent on performing both the overnight and the second, shorter trypsin digestion steps; omitting the second digestion reduces peptide yield and can bias the recovered peptide population toward more readily accessible cleavage sites. TMT (or TMTpro) labeling efficiency depends on maintaining the correct ratio of peptide to reagent and on room‐temperature incubation for the full hour specified; underlabeling leads to incomplete tag incorporation, which is not straightforward to distinguish from genuine low abundance during downstream quantification, and can therefore introduce spurious quantitative differences between channels if left uncontrolled.

In Basic Protocol 3, the choice of normalization strategy is a critical determinant of the final result: median scaling within each biological group, as implemented in the GroupScaling function, corrects for loading and labeling variation between channels without assuming that all proteins are equally abundant across conditions, which is the more common failure mode of quantile‐based methods when a small number of proteins dominate the total signal. A minimum of four biological replicates per condition should be used throughout, as using fewer replicates reduces statistical power to a degree that increases the risk of both false‐positive and false‐negative calls in the differential enrichment analysis, particularly for lower‐abundance interactors near the limit of detection.

### Troubleshooting

Common problems encountered during this protocol, their likely causes, and recommended solutions are listed in Table 4.

**Table 4:** Troubleshooting Guide for qPLEX‐RIME

Problem	Possible Cause	Solution
DSG solution shows visible precipitate	Incomplete dissolution in DMSO; DSG added too quickly to PBS	Discard and prepare a fresh DSG/DMSO stock; add dropwise under constant agitation with an orbital shaker.
Chromatin fragments too large after sonication (gel shows high‐molecular‐weight smear, Fig. 2)	Insufficient sonication cycles; low water level/ice in sonicator bath; lysate too viscous	Increase sonication cycles by 2‐3 rounds; confirm that the Bioruptor water bath is adequately filled and cooled; reoptimize cycle number for the specific cell line.
Chromatin over‐fragmented or signal lost after sonication	Excess sonication cycles; sample overheating during sonication	Reduce number of cycles; ensure samples remain on ice between sonication rounds; verify cooling water temperature.
Weak or absent bait protein signal on western blot after IP	Insufficient antibody amount; short antibody‐bead coupling time; low antibody specificity/affinity	Increase antibody amount per pulldown; extend coupling to 24 hr at 4°C rather than 3 hr at room temperature; confirm antibody performance by western blot before committing to a full experiment.
High nonspecific background/numerous contaminant proteins identified by MS	Insufficient bead washing; use of non‐LoBind tubes; no matched IgG control included	Increase number of RIPA washes; use Protein LoBind tubes throughout Basic Protocol 2; always run a matched IgG (or untagged) control alongside the target IP.
Low peptide yield after C18 clean‐up	Incomplete acidification before loading; column overloaded; incomplete elution	Confirm sample that pH is adjusted to <3 with formic acid before loading; match column capacity to peptide amount; verify elution volume follows manufacturer's recommendations.
Poor or uneven TMT/TMTpro labeling efficiency (missing or low‐intensity reporter channels)	TMT reagent hydrolyzed by moisture; incorrect peptide‐to‐label ratio; incubation time too short	Use fresh, single‐use TMT aliquots; keep reagent anhydrous until use; confirm exactly 1 hr room‐temperature incubation before quenching with hydroxylamine.
Poor chromatographic resolution or peak broadening during LC‐MS/MS	Column overloaded; high‐pH fractionation incomplete; gradient not optimized	Verify peptide load per fraction; confirm all nine high‐pH fractions were collected; reoptimize the elution gradient if peak shape is poor.
Low number of protein/peptide identifications in Proteome Discoverer	Incorrect precursor/fragment mass tolerances; outdated or mismatched FASTA database; poor spectral quality	Confirm SequestHT search parameters match those specified; verify that the UniProt FASTA matches the correct organism and release; check MS calibration and reacquire if spectral quality is poor.
Poor reproducibility between biological replicates (outliers on PCA plot, Supporting Information Fig. 8)	Unequal bait protein abundance between conditions; batch effects between processing days; incomplete normalization	Verify bait protein levels by western blot before pooling; process all conditions for a given replicate on the same day where possible; confirm that GroupScaling median normalization was applied correctly.
Few or no differentially enriched proteins identified	Fewer than the recommended 4 biological replicates; weak immunoprecipitation efficiency; incorrect contrast specification	Increase to a minimum of 4 biological replicates per condition; confirm IP efficiency by western blot; verify that the contrast formula in computeDiffStats() correctly reflects the intended comparison.

### Statistical Analysis

Differential protein enrichment between conditions is assessed using the limma‐based statistical framework implemented in qPLEXanalyzer (computeDiffStats, Step 9). Limma was originally developed for microarray data and applies an empirical Bayes procedure that moderates each protein's variance estimate toward a common, pooled value estimated across all proteins in the experiment (Smyth, 2004). This borrowing of information across proteins stabilizes variance estimates when the number of biological replicates is small, as is typically the case in qPLEX‐RIME experiments (a minimum of four replicates per condition, as noted under Critical Parameters), and produces a moderated *t*‐statistic that is more robust than a conventional per‐protein *t*‐test under these conditions. For each specified contrast (e.g., bait pulldown versus IgG control, or treated versus untreated), a linear model is fitted to the summed peptide intensities for each protein, giving a log_2_(fold change) and an associated moderated *p*‐value. *P*‐values are corrected for multiple testing using the Benjamini‐Hochberg method to control the false discovery rate. Proteins are considered significantly enriched at an adjusted *p*‐value <0.05 and an absolute log_2_(fold change) > 1; this is the threshold applied to Figure 7 and to Supporting Information Figure 9, and both criteria are shown for all identified proteins in those plots rather than for significant proteins alone, allowing readers to assess the full distribution of enrichment and significance rather than only the proteins passing threshold.

This approach assumes that most proteins in the dataset are not differentially enriched between conditions, an assumption that is generally reasonable for interactome data dominated by background binders, but that can be violated in experiments comparing conditions with substantially different global proteomes (e.g., primary tumor tissue versus cell lines), in which case the median‐based GroupScaling normalization (see the Data normalization section of Basic Protocol 3) becomes particularly important to satisfy this assumption before fitting the model. Alternative approaches include a standard (non‐moderated) two‐sample *t*‐test, which is more prone to inflated significance for proteins with spuriously low variance estimates when replicate numbers are small, and nonparametric methods such as the reproducibility‐optimized test statistic (ROTS) or rank‐based tests, which make fewer distributional assumptions but generally have lower statistical power at typical qPLEX‐RIME replicate numbers (*n* = 4‐6). For datasets with a high proportion of missing peptide intensities across channels, tools designed explicitly for missing‐value handling in quantitative proteomics, such as MSstats or DEqMS, may be considered as a complement or alternative to the limma‐based workflow described here.

### Understanding Results

Successful execution of Basic Protocol 1 is first assessed at the chromatin fragmentation step (step 19, Fig. 2): a 2% agarose gel or E‐Gel of reverse‐cross‐linked, purified chromatin should show a discrete smear centered between 200 and 300 bp, as seen for lanes B and C in Figure 2. A smear skewed toward higher molecular weight (lane A) indicates under‐sonication, which should be corrected by performing two or three additional cycles before proceeding, whereas a visible high‐molecular‐weight band (lane D) indicates absence of, or extremely inefficient, sonication. Efficient immunoprecipitation is best confirmed by western blotting for the bait protein comparing input, unbound, and bead‐eluate fractions; a successful pulldown should show clear depletion of the bait from the unbound fraction and strong enrichment in the eluate relative to a matched IgG control, and this comparison should also be used, as described under Strategic Planning, to confirm similar bait protein abundance across conditions before committing samples to the full qPLEX‐RIME workflow.

For Basic Protocol 2, the main indicator of success is peptide yield and labeling efficiency rather than a dedicated figure, as only small amounts of on‐bead‐digested material are generated at this stage; users should expect sufficient peptide material for downstream fractionation without a visible pellet or cloudiness in the eluate, and confirmation of successful outcome is only obtained once data is returned from the mass spectrometer in Basic Protocol 3.

The bioinformatic analysis in Basic Protocol 3 generates a series of quality‐control outputs that should be inspected in sequence before proceeding to differential analysis. The intensity distribution and boxplots (Fig. 4; Supporting Information Figs. 1 and 5) should show broadly similar median intensities and distributions across all samples; pronounced differences between samples before normalization are expected and are corrected by GroupScaling, so distributions should visibly tighten and align between Figures 4 and 6. The correlation plot (Supporting Information Figs. 2 and 6) should show consistently high correlation coefficients between biological replicates within the same group, and comparatively lower correlation between different groups (e.g., bait pulldown versus IgG control); replicates that fail to correlate as strongly as other members of their group are candidate outliers and should be investigated (see Troubleshooting) rather than automatically excluded. Hierarchical clustering (Supporting Information Figs. 3 and 7) and the PCA plot (Fig. 4; Supporting Information Fig. 8) provide a complementary view of the same relationships: samples should cluster, and separate along principal components, primarily by biological group rather than by processing batch or run order. If samples instead cluster by batch, this indicates a technical rather than biological source of variance that should be addressed, where possible, by including batch as a covariate in the statistical model. The coverage plot (Supporting Information Fig. 4) confirms enrichment efficiency directly: a successful bait pulldown should show peptide coverage distributed across a substantial proportion of the bait protein's sequence, and unexpectedly low or patchy coverage suggests a suboptimal immunoprecipitation even when downstream interactor data appear otherwise reasonable.

The MA plot (Supporting Information Fig. 9) and volcano plot (Fig. 7) are used to identify differentially enriched proteins from the final contrast (e.g., bait pulldown versus IgG control). As a positive control, the bait protein itself should appear among the most significantly and highly enriched points in the volcano plot; its absence from this position, even when other proteins show apparent enrichment, is a strong indicator of a failed or nonspecific pulldown and should prompt review of the western blot and coverage plot data before the remaining hits are interpreted biologically. Genuine interactors are expected to appear as a modest number of proteins with both high fold change and low adjusted *p*‐value, whereas the majority of identified proteins, representing background binders common to both bait and control pulldowns, should cluster near the plot's origin. Unanticipated findings, such as known interactors failing to reach significance, or the identification of proteins with no known association with the bait, are not necessarily indicative of failure and may reflect genuine condition‐specific or context‐specific interactome changes; these should be reported alongside the expected positive controls rather than omitted, and, where feasible, followed up by an orthogonal method such as co‐immunoprecipitation‐western blot for a subset of novel candidates.

Occasional replicates that appear as outliers on the correlation plot, hierarchical clustering, or PCA (e.g., an ER replicate clustering closer to the IgG group than to other ER replicates) should not be discarded by default. Such a pattern most commonly reflects a technical issue at the immunoprecipitation stage, most often insufficient bait protein abundance or antibody performance in that specific replicate (see Critical Parameters and Troubleshooting), and should first be cross‐checked against that replicate's western blot and coverage plot data before any decision is made. Where an outlier's IP efficiency is confirmed to be poor, exclusion should be reported explicitly, together with the criterion used; where no clear technical cause can be identified, we recommend retaining the replicate and relying on the moderated statistics described under Statistical Analysis, which are more robust than an unmoderated test to the influence of a single divergent replicate, rather than removing data post hoc without a defined justification.

### Time Considerations

Basic Protocol 1 (sample processing and immunoprecipitation) typically spans two working days. On Day 1, cross‐linking, quenching, and cell harvesting take ∼1 hr of hands‐on time, followed by 30‐45 min for the three‐step lysis (LB1‐LB3) and a further 1‐1.5 hr for sonication and gel‐based verification of fragment size (Fig. 2); antibody‐bead coupling can be run in parallel earlier the same day (3 hr at room temperature) or set up the day before (24 hr at 4°C). The immunoprecipitation itself is incubated overnight (∼12‐16 hr) with rotation in the cold room. On Day 2, bead washing (ten washes with RIPA plus two with AMBIC) takes ∼1‐1.5 hr, after which samples reach the first safe stopping point and can be snap‐frozen for later processing.

Basic Protocol 2 (proteomic processing) requires a further 2‐3 days, largely driven by incubation rather than hands‐on time. On‐bead digestion begins with an overnight trypsin incubation (∼16 hr) followed by a second 4‐hr digestion the next day; acidification and C18 peptide clean‐up take ∼1‐1.5 hr, after which samples can again be stored at –20°C. TMT or TMTpro labeling requires only 1 hr at room temperature, but pooling and drying the combined sample by centrifugal concentration can take 2‐3 hr depending on starting volume, after which the labeled pool is again stable in storage. High‐pH fractionation into nine fractions takes ∼2‐3 hr of hands‐on time. LC‐MS/MS acquisition of all nine fractions using the 120‐min gradient described in step 12 requires roughly 18 hr of largely unattended instrument time, and is typically run overnight.

Basic Protocol 3 (bioinformatic analysis) is the most variable in duration, depending on user familiarity with R. Preparing the protein‐to‐gene mapping file from UniProt (steps 1‐3) takes 30‐60 min. For a user already familiar with R and RStudio, loading of the data, quality control, normalization, protein summarization, and differential statistical analysis (steps 4‐15) can typically be completed within a single working day (4‐6 hr), including generation of all diagnostic plots. Users less experienced with R should allow 1‐2 days to accommodate troubleshooting of file formats, package installation, and interpretation of the quality‐control outputs described under Understanding Results.

In total, from cell harvest to a completed differential enrichment table, qPLEX‐RIME typically requires 5‐7 working days, the majority of which is unattended incubation or instrument time rather than active hands‐on work.

### Author Contributions

**Soleilmane Omarjee**: Investigation; supervision; validation; writing—review and editing. **Evangelia K. Papachristou**: Conceptualization; investigation; methodology; validation; writing—review and editing. **Chandra Sekhar Reddy Chilamakuri**: Data curation; formal analysis; methodology; software; visualization; writing—review and editing. **Hamish McMillan**: Validation; writing—review and editing. **Kamal Kishore**: Data curation; formal analysis; methodology; software; visualization; writing—review and editing. **Joshua Kent**: **Valar Nila Roamio Franklin**: Methodology; validation; writing—review and editing. **Clive D'Santos**: Conceptualization; investigation; project administration; supervision; writing—review & editing. **Jason S. Carroll**: Conceptualization; funding acquisition; investigation; project administration; resources; supervision; writing—review and editing.

### Conflict of Interest

The authors declare no conflicts of interest.

### AI Use Statement

During preparation of this manuscript, the authors used Claude (Anthropic) under Enterprise Licence solely to check and correct English language, grammar, and sentence structure in text that was fully authored by the human researchers. The tool was not used to generate original content, ideas, data, or interpretations. All figures were produced manually by the authors using code provided in the manuscript, with no generative AI involvement.

## Supporting information




*Supporting Information Figures S1‐S11*.

## Data Availability

The mass spectrometry proteomics data generated using the qPLEX‐RIME method described in this protocol, and analyzed in Papachristou et al. (2018), have been deposited to the ProteomeXchange Consortium via the PRIDE partner repository under dataset identifier PXD007968 (https://www.ebi.ac.uk/pride/archive/projects/PXD007968). Readers applying this protocol to their own samples are encouraged to deposit their resulting raw and processed mass spectrometry data in PRIDE/ProteomeXchange, following the data‐sharing policy of the journal in which their findings are published.
